# Hypertrophy Stimulation at the Onset of Type I Diabetes Maintains the Soleus but Not the EDL Muscle Mass in Wistar Rats

**DOI:** 10.3389/fphys.2017.00830

**Published:** 2017-10-26

**Authors:** Marco A. S. Fortes, Maria V. M. Scervino, Gabriel N. Marzuca-Nassr, Kaio F. Vitzel, Carlos H. da Justa Pinheiro, Rui Curi

**Affiliations:** ^1^Department of Physiology and Biophysics, Institute of Biomedical Sciences, University of São Paulo, São Paulo, Brazil; ^2^Department of Internal Medicine, Faculty of Medicine, Universidad de La Frontera, Temuco, Chile; ^3^School of Health Sciences, College of Health, Massey University, Albany, New Zealand; ^4^Interdisciplinary Post-Graduate Program in Health Sciences, Cruzeiro do Sul University, São Paulo, Brazil

**Keywords:** skeletal muscle, protein synthesis, muscle strength, hyperglycemia, electrostimulation, muscle mass regulation

## Abstract

Diabetes mellitus induces a reduction in skeletal muscle mass and strength. Strength training is prescribed as part of treatment since it improves glycemic control and promotes increase of skeletal muscle mass. The mechanisms involved in overload-induced muscle hypertrophy elicited at the establishment of the type I diabetic state was investigated in Wistar rats. The purpose was to examine whether the overload-induced hypertrophy can counteract the hypotrophy associated to the diabetic state. The experiments were performed in oxidative (soleus) or glycolytic (EDL) muscles. PI3K/Akt/mTOR protein synthesis pathway was evaluated 7 days after overload-induced hypertrophy of soleus and of EDL muscles. The mRNA expression of genes associated with different signaling pathways that control muscle hypertrophy was also evaluated: mechanotransduction (FAK), Wnt/β-catenin, myostatin, and follistatin. The soleus and EDL muscles when submitted to overload had similar hypertrophic responses in control and diabetic animals. The increase of absolute and specific twitch and tetanic forces had the same magnitude as muscle hypertrophic response. Hypertrophy of the EDL muscle from diabetic animals mostly involved mechanical loading-stimulated PI3K/Akt/mTOR pathway besides the reduced activation of AMP-activated protein kinase (AMPK) and decrease of myostatin expression. Hypertrophy was more pronounced in the soleus muscle of diabetic animals due to a more potent activation of rpS6 and increased mRNA expression of insulin-like growth factor-1 (IGF-1), mechano-growth factor (MGF) and follistatin, and decrease of myostatin, MuRF-1 and atrogin-1 contents. The signaling changes enabled the soleus muscle mass and force of the diabetic rats to reach the values of the control group.

## Introduction

Reduced protein synthesis stimulation and increased protein degradation (Sandri, 2008; Schiaffino et al., 2013) are associated with the loss of skeletal muscle mass in type 1 diabetes (Barazzoni et al., 2004). Animals with diabetes mellitus induced by streptozotocin administration have increased short-term proteolytic activity in the skeletal muscle (1–3 days after diabetes induction) that returns to control values after 5–10 days (Pepato et al., 1996). The increase in short-term myostatin expression is associated with the severity of skeletal muscle loss, which is abolished by insulin treatment (Chen et al., 2009).

The mechanisms associated with sarcopenia and consequently on its prevention have been investigated (Roden, 2015). Streptozotocin-induced diabetic animals exhibit increased AMP kinase (AMPK) phosphorylation (Vitzel et al., 2013b) and impairs muscle hypertrophy through a reduced activation of protein synthesis signaling such as protein kinase B (Akt) in Ser^473^, mammalian target of rapamycin (mTOR) in Ser^2448^, ribosomal protein S6 (rpS6) in Thr^389^, and 4E binding protein-1 (4EBP-1) in Thr^37^ (Bolster et al., 2002; Thomson and Gordon, 2005).

Physical exercise is prescribed for diabetic patients in order to improve glycemic control. However, little is known about the association of diabetes mellitus and the skeletal muscle mass hypertrophy induced by resistive physical exercise. The overload due to ablation of the synergistic muscles is the most used experimental approach for the study of the mechanisms involved in the skeletal muscle hypertrophy (Rosenblatt and Parry, 1992; Phelan and Gonyea, 1997; Bodine et al., 2001; Ishido et al., 2006; Katta et al., 2010; Paturi et al., 2010). Peak activation of protein synthesis pathway is described after 7 days of the synergistic ablation surgery. After 30 days of hypertrophic stimulus, the skeletal muscle mass increase reaches a plateau (Armstrong and Ianuzzo, 1977; Farrell et al., 1999; Katta et al., 2010).

Diabetic myopathy has several consequences for skeletal muscle (for review see Krause et al., 2011; D'Souza et al., 2013) such as reduction in physical abilities, muscle mass, and strength (Andersen et al., 1996, 1997, 2004). In fact, only 3 days after diabetes induction by streptozotocin injection in rats, a significant reduction in soleus, EDL, and gastrocnemius muscle mass is described (Pepato et al., 1996; Price et al., 1996). However, despite the exacerbated muscular hypotrophy in the diabetic state, the ability of the soleus and extensor digitorum longus (EDL) muscles to respond to a hypertrophic stimulus, even under uncontrolled hyperglycemic state, is not impaired in streptozotocin-induced diabetic rats (Fortes et al., 2015).

Taking into account the rapid onset of muscle loss upon diabetes induction and the prompt hypertrophic response caused by overload, we investigated if a hypertrophic stimulation could prevent or attenuate the muscle mass loss induced by the onset of the type 1 diabetes. For this purpose, the effects of tibialis anterior muscle ablation and gastrocnemius muscle tenotomy and the consequent overload-induced hypertrophy of the EDL and soleus muscles, respectively, concomitantly with the establishment of the diabetic state, were investigated in rats. We examined and compared the effects of short-term diabetic condition associated with a state of intense skeletal muscle stimulus for hypertrophy induced by overload on signaling pathways associated with protein synthesis and degradation controlled by phosphoinositide 3-kinase (PI3K)-Akt-mTOR, including the E3 ubiquitin ligase that are increased in atrophic conditions, MuRF-1 and atrogin-1; mechanotransduction by rpS6 phosphorylation and mRNA expression of Focal Adhesion Kinase (FAK) and MGF; mRNA expressions of myostatin and follistatin, Wnt/β-catenin and MG53 that are involved in myogenesis and may affect insulin signaling (Jung and Ko, 2010; Lee et al., 2010). The purpose of our study is to investigate the pathways associated to overload-induced muscle hypertrophy and to examine whether the hypertrophy would counteract the hypotrophy occurring soon after the onset of type I diabetes.

The hypothesis of the present study is that different signaling pathways are activated during skeletal muscle overload in type 1 diabetes mellitus in order to overcome the consequences of the low plasma insulin levels and so prevent/attenuate the marked loss of skeletal muscle mass observed after the onset of the diabetic state.

## Methods

### Animals and diabetes induction protocol

Type 1 diabetes was induced in male Wistar rats weighing 200 ± 50 g obtained from the Institute of Biomedical Sciences at the University of São Paulo. The animals were housed in groups of three with water and food provided *ad libitum* in a room with a 12/12-h light/dark cycle at 22°C. Protocols for all experimental procedures were approved by the animal ethics committee (CEUA-ICB-USP, 26/16/03) and performed according with the Guide for the Care and Use of Laboratory Animals (Institute of Laboratory Animal Resources, National Academy of Sciences, Washington, DC, USA) and the principles of the Brazilian College of Animal Experimentation (COBEA).

Hyperglycemic condition similar to type 1 diabetes was achieved by injection of 65 mg/kg b.w. streptozotocin dissolved in citrate buffer (pH 4.5) into the caudal vein (Ungvari et al., 1999; Fortes et al., 2015) whereas only the control group received the same volume of citrate buffer. After a 24-h period of the streptozotocin injection, blood glucose levels confirmed the diabetic state and only animals with or above 400 mg/dL (22.2 mmol/L) were used in the experiments. Animals displaying sustained hyperglycemia for 3 consecutive days (confirmation of the diabetic state) were submitted to surgery for skeletal muscle overload induction. Also, glucose levels were measured weekly and before tissue collection to ensure that the animals remained diabetic up to the end of the experiments as previously described (Fortes et al., 2015, 2016).

### EDL and soleus muscles overload protocol

Soleus muscle hypertrophy was induced by unilateral synergistic tenotomy of the gastrocnemius muscle as previously described (Goldberg, 1972; Armstrong et al., 1979; Owino et al., 2001; Fortes et al., 2015). EDL muscle overload was induced by unilateral synergistic ablation of the tibialis anterior muscle, and it was performed in a different set of animals, as previously described (Rosenblatt and Parry, 1992; Hamilton et al., 2010; Fortes et al., 2015).

Muscle overloading was kept during 7 days for evaluation of protein content and mRNA expression because muscle protein synthesis is at its peak under this condition (Baillie and Garlick, 1991). In another set of animals, 30 days of overload was employed for evaluation of fiber cross-sectional area (CSA), twitch and tetanic forces, muscle mass and twitch contractile properties. After 30 days of overload, hypertrophied muscle no longer has increased protein synthesis activity and trophic response has reached its peak, therefore, being an appropriate time point for measurements of contractile activity (Armstrong and Ianuzzo, 1977; Farrell et al., 1999; Katta et al., 2010). The unilateral ablation and tenotomy allows paired comparison between sham and overloaded muscles and avoid inaccuracies due to the use of different animals (Thomson and Gordon, 2006). By the end of the experimental protocol, the control (CTRL), and diabetic (DM) groups were obtained, each one with a contralateral limb that does not undergo surgical intervention, the sham (CL), and the other limb that was ablated or tenotomized for hypertrophy (H).

### Analysis of skeletal muscle contractile function and *in situ* electrical stimulation

Evaluations of skeletal muscle contractile function were performed as previously described (Pinheiro et al., 2012; Fortes et al., 2015). After being anesthetized by using intraperitoneal injection of ketamine and xylazine (90 and 10 mg/kg b.w., ip., respectively), the rats were fixed on an acrylic platform and the hindlimb skin was excised for the assessment of the sciatic nerve and a platinum electrode was placed at the nerve. The synergistic and antagonist muscles were tenotomized to avoid interference in the measurements. The resting length of soleus or EDL muscles was adjusted to obtain maximum tension (the ankle joint approximately at a 90° angle) upon stimulation using the MultiStim System D330 electrical stimulation device (Digitimer Ltd, Welwyn Garden City, Hertfordshire, UK) by traction regulation of the hook coupled to the distal tendon and the force transducer (Grass Technologies, West Warwick, RI). Five twitch contractions were employed for determination of the muscle twitch force, time to peak (TTP—time between the onset of force development until peak tension), half relaxation time (HRT—time of muscle relaxation half-way from peak tension), late relaxation time (LRT—time of muscle relaxation between 50 and 25% of peak tension), and rate of force development (RFD—amount of force generated per time unit during skeletal muscle contraction). The twitch stimulus consisted of 500 μs pulse duration at 1 Hz with adjusted voltage to produce maximum force. For determination of the tetanic force, 10 successive contractions were elicited at 100 Hz, with 2 s duration each and 10 s of recovery period between them, being the first tetanic contraction used to assess the maximum tetanic force. Data were recorded using the AqDados® software (version 4.16, Lynx Tecnologia Eletrônica Ltda, São Paulo, Brazil) and analyzed using AqAnalysis® software (version 4.16, Lynx Tecnologia Eletrônica Ltda, São Paulo, Brazil).

### Histological analysis

Evaluation of fiber CSA was performed as previously described (Bodine and Baar, 2012; Marzuca-Nassr et al., 2016). Images were taken using an optical microscope (Nikon Eclipse E1000, Fukuoka, Japan) attached to a digital camera (NixonDXM1200). The images were analyzed using the AxioVision® program (version 4.8.1.0, Carl Zeiss Imaging Solutions, Jena, Germany).

### Western blot

Proteins were extracted and assayed as previously described (Vitzel et al., 2013a; Fortes et al., 2015; Marzuca-Nassr et al., 2016). The primary antibodies used were: p-Akt at Ser 473 (9271), Akt (9272), p-rpS6 at Ser 240/244 (5364), rpS6 (2217), p-4EBP-1 at Thr 37/46 (2855), 4EBP-1 (9644), p-AMPK-α at Thr 172 (2535), AMPK- α (2532) from Cell Signaling Technology (Beverly, MA, USA), and atrogin-1 (AP2041) and muscle RING-finger protein-1 (MuRF-1) (MP3401) from ECM Biosciences (Versailles, KY, USA). Cell Signaling Technology (Beverly, MA, USA) provided the secondary antibody conjugated to peroxidase. The images were captured using an Amersham Imager 600 (Amersham/GE Healthcare, Little Chalfont, UK). The densitometry of the specific bands was measured using the ImageJ software (NIH, Bethesda, MD, USA). Results were normalized to the total loading of protein in each sample, quantified by Ponceau S staining (Romero-Calvo et al., 2010; Fortes et al., 2016). The contralateral muscle was considered as 1. Western blotting gels and ponceau staining are presented as Supplementary Material.

### Real-time PCR

Total RNA was extracted according to the manufacturer's specification using the RNeasy kit (QIAGEN, Hilden, Germany). RNA concentration was estimated based on the optical density of the samples at 260 nm obtained in a DS-11 FX spectrophotometer (DeNovix, Wilmington, DE, USA). One microgram of RNA was used for reverse transcription and cDNA synthesis, using oligo-dT primers and the RevertAid M-MuLV reverse transcriptase kit (Invitrogen/Life Technologies, Carlsbad, CA, USA), following the manufacturer's instruction. The amplification of target genes sequences in the cDNA was performed through utilization of a SYBR Green qPCR kit (Invitrogen/Life Technologies, Carlsbad, CA, USA) together with specific sense and antisense primers (Exxtend, São Paulo, Brazil) (Table 1) performed in a Rotor Gene 6000 equipment (Corbett Research, Mortlake, Australia). Relative gene expression calculation was performed by 2^−ΔΔCT^ (Livak and Schmittgen, 2001). Results were normalized to the expression of hypoxanthine phosphoribosyltransferase 1 (HPRT1).

**Table 1:** Primer sequences used for the Real-time PCR assays.

	**Primer sense**	**Primer antisense**
FAK	5′-AAGGAGCACCTCTCAAACCG-3′	5′-CATCGCTCCGACAGCATTTG-3′
Akt1	5′-GCCCAAGCACCGTGTGACCA-3′	5′-GCGACCTGTGGCCTTCTCCT-3′
mTOR	5′-AGGGACCACTGTGCCAGAATCCA-3′	5′-TGAGAGAAATCCCGACCAGTGAGC-3′
β-Catenin	5′-AGCGCTGGTGAAAATGCTTG-3′	5′-CGCACTGCCATTTTAGCTCC-3′
MuRF-1	5′-GGACCGGCATGGGGTGTACG-3′	5′-TTTCTGCAGGGGCCGACTGG-3′
Atrogin-1	5′-CGGCACCTTCGTGAGCGACC-3′	5′-GTGCAGATATCCATGGCGCTCCT-3′
MG53	5′-CAGGCGCTAAGCACTAACCT-3′	5′-GGTCCTGCTCGCAGTAGATG-3′
IGF-1	5′-TGAGCGCACCTCCAATAAAGA-3′	5′-GAACTGAAGAGCGTCCACCA-3′
MGF	5′-TGGTGGACGCTCTTCAGTTC-3′	5′-TCCGGAAGCAACACTCATCC-3′
Myostatin	5′-TACCACGGAAACAATCATTACCAT-3′	5′-TGCCATCCGCTTGCATT-3′
Follistatin	5′-AGCGAGTGTGCCATGAAG-3′	5′-GAGTGGAAGAGATAGGGAAGC-3′
Wnt 7a	5′-GCGCTCTAGGACAGTCTCCA-3′	5′-GGGGCAATCCACATAGCCTG-3′
Axin 2	5′-CTCAGCAAAAAGGGAAATTACAGGTAT-3′	5′-ACTGTCTCGTCGTCCCAGATCTC-3′
HPRT1	5′-GCGAAAGTGGAAAAGCCAAGT-3′	5′-GCCACATCAACAGGACTCTTGTAG-3′

*FAK, Focal Adhesion Kinase; Akt1, Protein Kinase B; mTOR, Mammalian target of rapamicin; MuRF-1, Muscle RING-Finger protein-1; MG53, Mitsugumin 53; IGF-1, Insulin-like growth factor 1; MGF, Mechano growth factor; Wnt 7a, Wingless-Type MMTV Integration Site Family, Member 7A; HPRT1, Hypoxanthine Phosphoribosyltransferase 1*.

### Statistical analysis

Statistical analysis was performed using the GraphPad Prism® software (version 4.01; El Camino Real, CA, USA). Results are presented as mean ± standard error of the mean (SEM) and were analyzed by two-way analysis of variance (ANOVA) followed by the Bonferroni post-test (for comparison between three or more groups). Differences were considered statistically significant for *p* < 0.05. CSA of the EDL and soleus muscles fibers were not normally distributed, therefore, difference was considered significant when there was no overlap between the 95% confidence interval of the median calculated for each experimental group (Gehrig et al., 2008; Fortes et al., 2015; Marzuca-Nassr et al., 2016).

## Results

### Body weight and glycaemia

**Figure 1:**
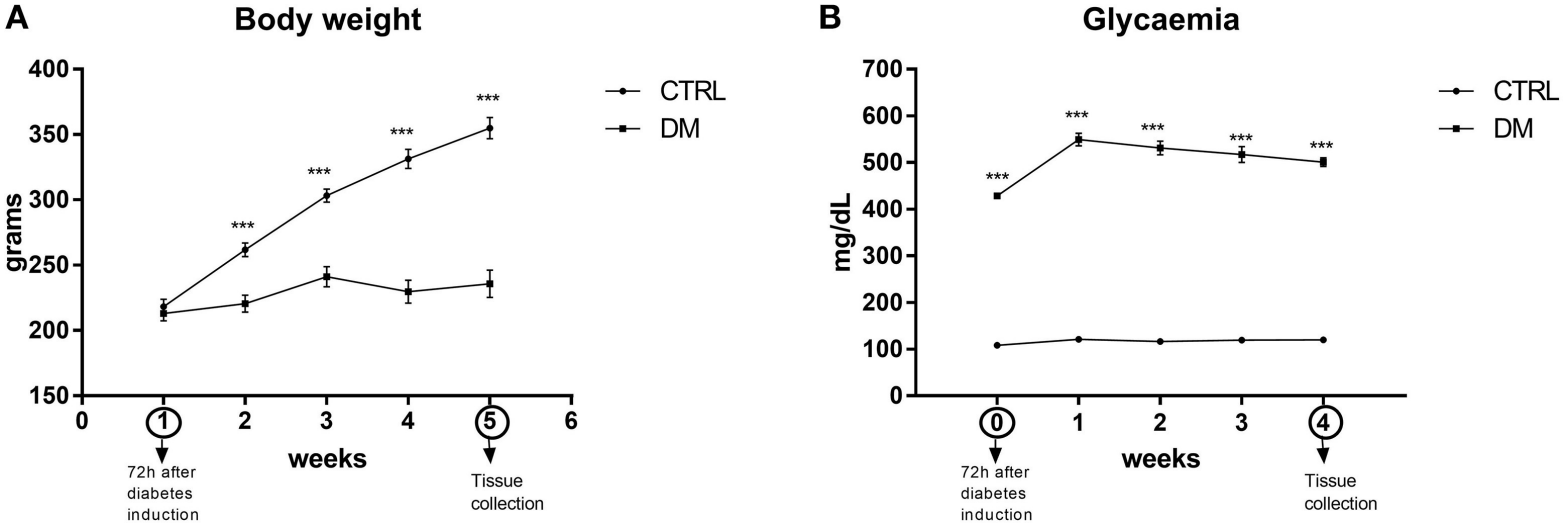
Body weight **(A)** and glycaemia **(B)** of control and diabetic rats during 4 weeks of the experiments. Results are expressed as mean ± SEM of 13 animals. ^***^p < 0.001, using repeated measures two-way ANOVA followed by Bonferroni post-test. CTRL, control group; DM, diabetic group.

The body mass of the control animals increased regularly whereas in diabetic animals remained almost unchanged for 30 days (Figure 1). Glycaemia remained unchanged in controls whereas in diabetic rats it was 3.9-fold higher than in the control group 72 h after induction of diabetes and further increased by 17% over the 30-day period.

### Muscle mass, strength, and contractile properties

After 30 days of overload, the absolute and relative tetanic forces of the hypertrophied EDL muscle were different between diabetic and control animals. Absolute tetanic force increased by 40% in control and by 102% in diabetic rats (Table 2). EDL absolute twitch force was increased in the control and diabetic groups by 79 and 114%, respectively (Table 2).

**Table 2:** EDL mass, strength, and contractile properties after 30 days of overload.

**Measurements**	**Control group**		**Diabetic group**	
	**Hypertrophy**	**Contralateral**	**Hypertrophy**	**Contralateral**
**EDL MUSCLE**				
Wet weight (mg)	214.23 ± 11.16^aa^	171 ± 5.06	105.7 ± 9.09^a^ ^bbb^	76.17 ± 4.5^bbb^
Dry weight (mg)	57.03 ± 2.92^aa^	45.41 ± 1.2	28.8 ± 2.38^a^ ^bbb^	22 ± 1.75^bbb^
Wet weight per tibia length (mg/mm)	4.96 ± 0.26^aa^	3.94 ± 0.12	2.63 ± 0.22^a^ ^bbb^	1.97 ± 0.11^bbb^
Dry weight per tibia length (mg/mm)	1.32 ± 0.07^aa^	1.05 ± 0.03	0.71 ± 0.05^a^ ^bbb^	0.52 ± 0.05^bbb^
Absolute Tetanic Force (mN)	5132.29 ± 669.18^a^	3667.16 ± 410.77	3970.99 ± 496.61^a^	1962.24 ± 369.08
Specific Tetanic Force (mN/mg)	118.9 ± 35.94	103.03 ± 26.02	79.27 ± 25.91	49.7 ± 16.35
Absolute Twitch Force (mN)	1379.29 ± 196.61^a^	769.77 ± 97.51	1214.26 ± 165.12^a^	567.16 ± 124.38
Specific Twitch Force (mN/mg)	37.81 ± 9.56	22.25 ± 6.09	26.32 ± 7.43	15.24 ± 6.34
TTP (ms)	20 ± 0.02	22.85 ± 1.8	21.6 ± 1.6	24.0 ± 2.4
HRT (ms)	21.47 ± 1.7	20.3 ± 2.1	16.1 ± 1.6	17.3 ± 3.0
LRT (ms)^c^	10.9 ± 0.7	13.9 ± 2.3	8.5 ± 0.8	7.6 ± 0.8
RFD (mN/ms)^dd^	72.4 ± 10.9	41.1 ± 10.5	56.5 ± 11.1	22.9 ± 8.3

*Results are expressed as mean ± SEM of at least six animals. Data were analyzed using two-way ANOVA followed by Bonferroni post-test*.

a *p < 0.05*,

aa p < 0.01 CTRL-CL vs. CTRL-H and DM-CL vs. DM-H;

bbb p < 0.001 CTRL-CL vs. DM-CL and CTRL-H vs. DM-H;

c p < 0.05 CTRL vs. DM using two-way ANOVA only;

dd *p < 0.01 CTRL-CL/DM-CL vs. CTRL-H/DM-H using two-way ANOVA only. CTRL, Control group; DM, Diabetic group; CL, Contralateral; H, Hypertrophy; TTP, time to peak; HRT, half relaxation time; LRT, late relaxation time; RFD, rate of force development*.

In the EDL muscle of control animals, there was an increase of 25% in both absolute and normalized muscle wet weight due to the overload. In diabetic animals, there was an increase of 38 and 33% in the wet weight and the wet weight normalized by the tibia length, respectively (Table 2). In the control animals, there was an increase of 25% in the dry weight and the dry weight normalized by the tibia length. In diabetic animals, there was a 31 and 37% increase in dry weight and dry weight normalized by tibia length, respectively (Table 2).

The EDL muscle of diabetic rats had a decrease (by 45%) in LRT in the contralateral and in the hypertrophied (by 22%) EDL muscle when compared with control (Table 2). The RFD was higher in all hypertrophied muscles. In controls, there was an increase of 76% in relation to the contralateral muscle, whereas in the diabetic group it was raised by 2.5-fold.

After 30 days of overload, the absolute tetanic force of the hypertrophied soleus was different between the hypertrophied and contralateral muscles in both groups. Absolute tetanic force was increased by 77% in control animals and by 43% in diabetic rats (Table 3). The absolute twitch force of the soleus muscle was increased in the control and diabetic groups by 31 and 35%, respectively (Table 3).

**Table 3:** Soleus mass, strength, and contractile properties after 30 days of overload.

**Measurements**	**Control group**		**Diabetic group**	
	**Hypertrophy**	**Contralateral**	**Hypertrophy**	**Contralateral**
**SOLEUS MUSCLE**				
Wet weight (mg)	211.51 ± 10.34^aaa^	152.37 ± 7.74	156.12 ± 9.67^aaa bbb^	109.89 ± 7.72^bb^
Dry weight (mg)	51.58 ± 2.64^aaa^	40.05 ± 1.86	40.43 ± 1.6^aaa^ ^bbb^	27.75 ± 1.53^bbb^
Wet weight per tibia length (mg/mm)	4.96 ± 0.21^aaa^	3.58 ± 0.17	3.9 ± 0.22^aaa^ ^bb^	2.74 ± 0.18^b^
Dry weight per tibia length (mg/mm)	1.21 ± 0.05^aaa^	0.94 ± 0.04	1.01 ± 0.03^aaa^ ^bb^	0.69 ± 0.05^bbb^
Absolute Tetanic Force (mN)	4838.04 ± 379.19^aaa^	2729.97 ± 177.59	4571.73 ± 503.6^a^	3193.56 ± 341.29
Specific Tetanic Force (mN/mg)	93.41 ± 6.92	70.36 ± 7.08	97.57 ± 16.7	103.43 ± 8.57
Absolute Twitch Force (mN)^cc^ ^d^	1825.7 ± 217.96	1397.92 ± 166.21	1293.44 ± 52.34	956.23 ± 102.21
Specific Twitch Force (mN/mg)	35.17 ± 3.94	35.53 ± 4.3	30.24 ± 2.38	31.07 ± 2.61
TTP (ms)	25 ± 2.22	20 ± 0.0	21.7 ± 1.7	21.7 ± 1.7
HRT (ms)	5.0 ± 1.1	5.8 ± 1.1	4.5 ± 1.0	6.3 ± 1.5
LRT (ms)	3.5 ± 0.6^a^	7.9 ± 0.5	4.9 ± 1.4	6.0 ± 1.4
RFD (mN/ms)^d^	74.4 ± 7.3	63.1 ± 9.2	54.6 ± 7.7	45.2 ± 5.9

*Results are expressed as mean ± SEM of at least six animals. Data were analyzed using two-way ANOVA followed by Bonferroni post-test*.

a *p < 0.05*,

aaa p < 0.001 CTRL-CL vs. CTRL-H and DM-CL vs. DM-H;

b *p < 0.05*,

bb *p < 0.01*,

bbb p < 0.001 CTRL-CL vs. DM-CL and CTRL-H vs. DM-H;

cc p < 0.01 CTRL vs. DM using two-way ANOVA only;

d *p < 0.05 CTRL-CL/DM-CL vs. CTRL-H/DM-H using two-way ANOVA only. CTRL, Control group; DM, Diabetic group; CL, Contralateral; H, Hypertrophy; TTP, time to peak; HRT, half relaxation time; LRH, late relaxation time; RFD, rate of force development*.

Soleus muscle also presented an increase of 38% in the wet weight and the wet weight normalized by tibia length in the control group. In the diabetic group, there was an increase of 42 and 48% of the wet weight and the wet weight normalized by the tibia length, respectively (Table 3). In the control animals, the dry weight and the dry weight normalized by the tibia length were increased by 28% upon hypertrophy. In diabetic animals, there was an increase of about 45% in the dry weight and the dry weight normalized by the tibia length (Table 3).

The soleus muscle of control animals had a decrease in the LRT of 56% when they underwent hypertrophy (Table 3). The RFD was lower in the diabetic group both in the control and hypertrophied muscles.

### CSA of the EDL and soleus muscles fibers

**Figure 2:**
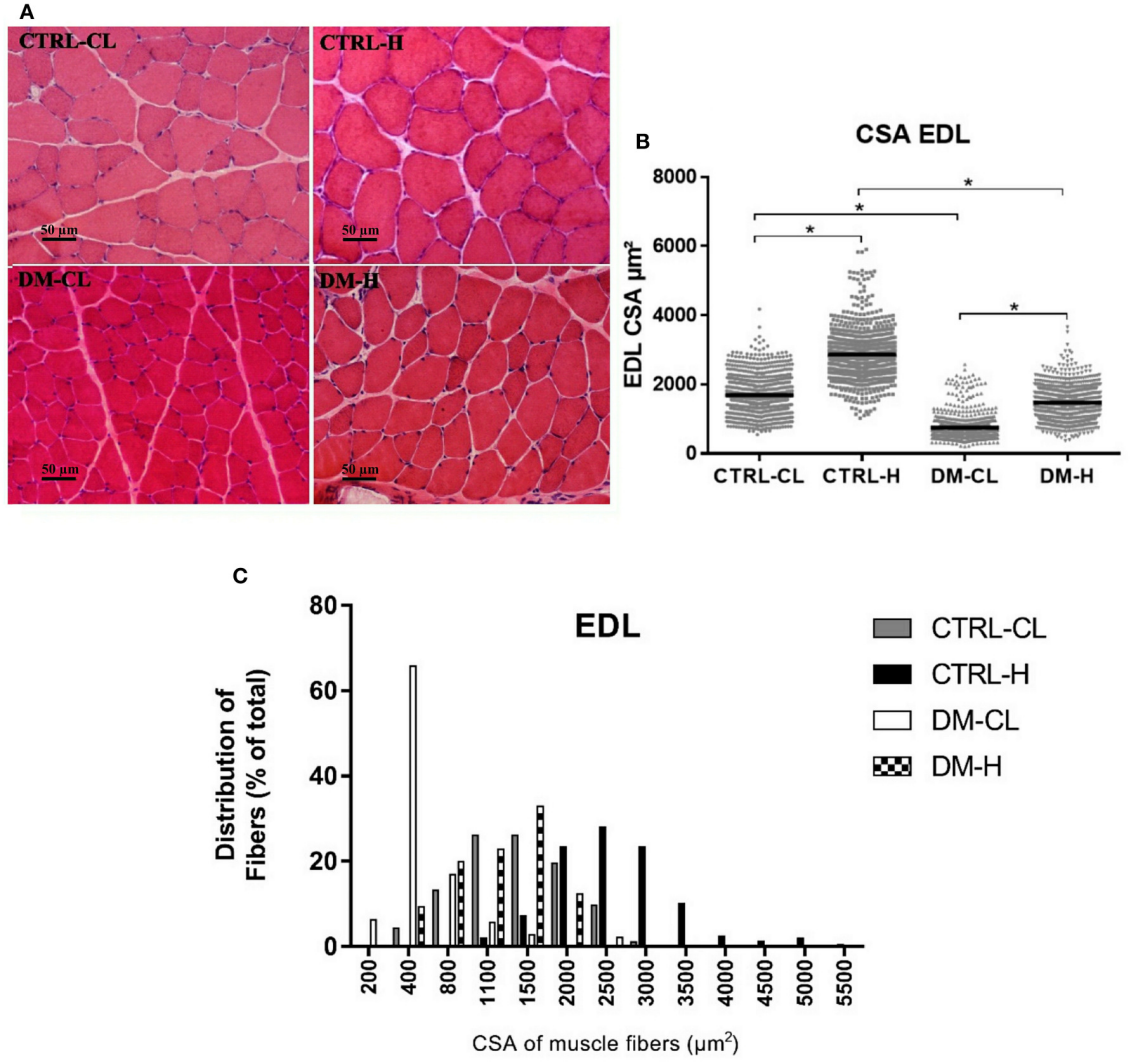
Cross-sectional areas (CSA) of the EDL muscle fibers. **(A)** Representative histological hematoxylin and eosin staining images of EDL muscle CSA. Reference bar represents 50 μm. **(B)** Dispersion graph of EDL muscle fibers CSA. **(C)** Frequency distribution of the EDL muscle fibers according to CSA ranges: 0–5,500 μm^2^. The results were analyzed as previously described using the 95% confidence interval of the median. ^*^Significant different considering the 95% confidence interval of the median. CTRL-CL, control group, contralateral muscle; CTRL-H, control group, hypertrophied muscle; DM-CL, diabetic group, contralateral muscle; DM-H, diabetic group, hypertrophied muscle.

**Figure 3:**
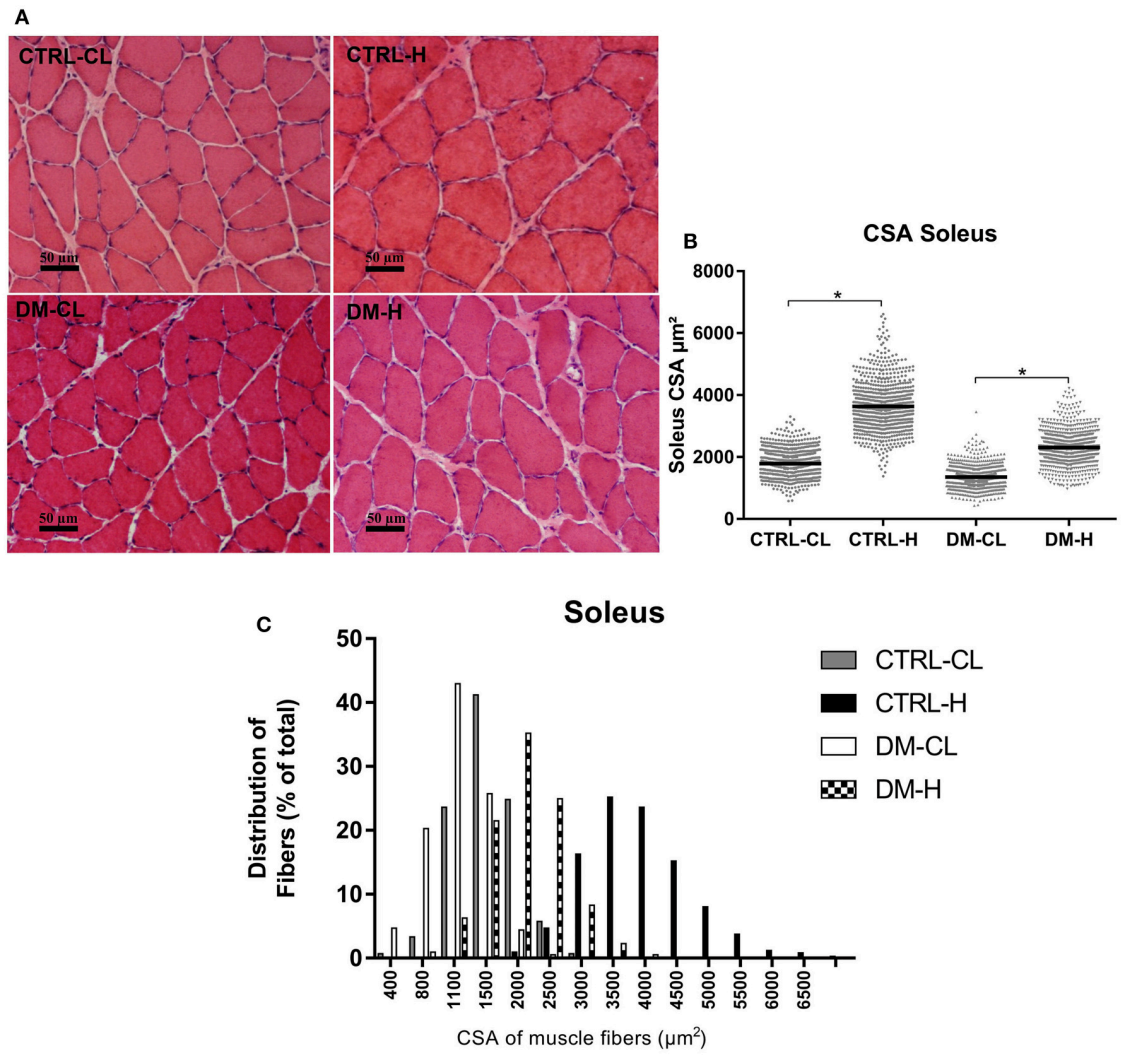
Cross-sectional areas (CSA) of the soleus muscle fibers. **(A)** Representative histological hematoxylin and eosin staining images of the soleus muscle CSA. Reference bar represents 50 μm. **(B)** Dispersion graph of soleus muscle fibers CSA. **(C)** Frequency distribution of the soleus muscle fibers according to the CSA ranges: 0–7,000 μm^2^. The results were analyzed as previously described using the 95% confidence interval of the median. ^*^Significant difference considering the 95% confidence interval of the median. CTRL-CL, control group, contralateral muscle; CTRL-H, control group, hypertrophied muscle; DM-CL, diabetic group, contralateral muscle; DM-H, diabetic group, hypertrophied muscle.

CSA of the EDL and soleus muscles fibers were markedly increased due to overload in all groups as compared to non-hypertrophied muscles (Figures 2A,B, 3A,B). This effect, quantitatively assessed by the 95% confidence interval of the median, was supported by qualitative analysis of the proportion of fibers in different ranges of CSA (Figures 2C, 3C). Frequency distribution (Figures 2C, 3C) was calculated and expressed as previously performed by others (Baehr et al., 2011; Ge et al., 2011; Kulakowski et al., 2011; Pistilli et al., 2011; Watson et al., 2012; Callahan et al., 2014; Marzuca-Nassr et al., 2016). In the EDL muscle of the control group there was a 69% increase and in the diabetic animals of 97% (Figure 2). In the soleus muscle, there was an increase of 103% in the control group and of 70% in the diabetic group (Figure 3).

### Modulation of the signaling protein contents after 7 days of muscle overload

**Figure 4:**
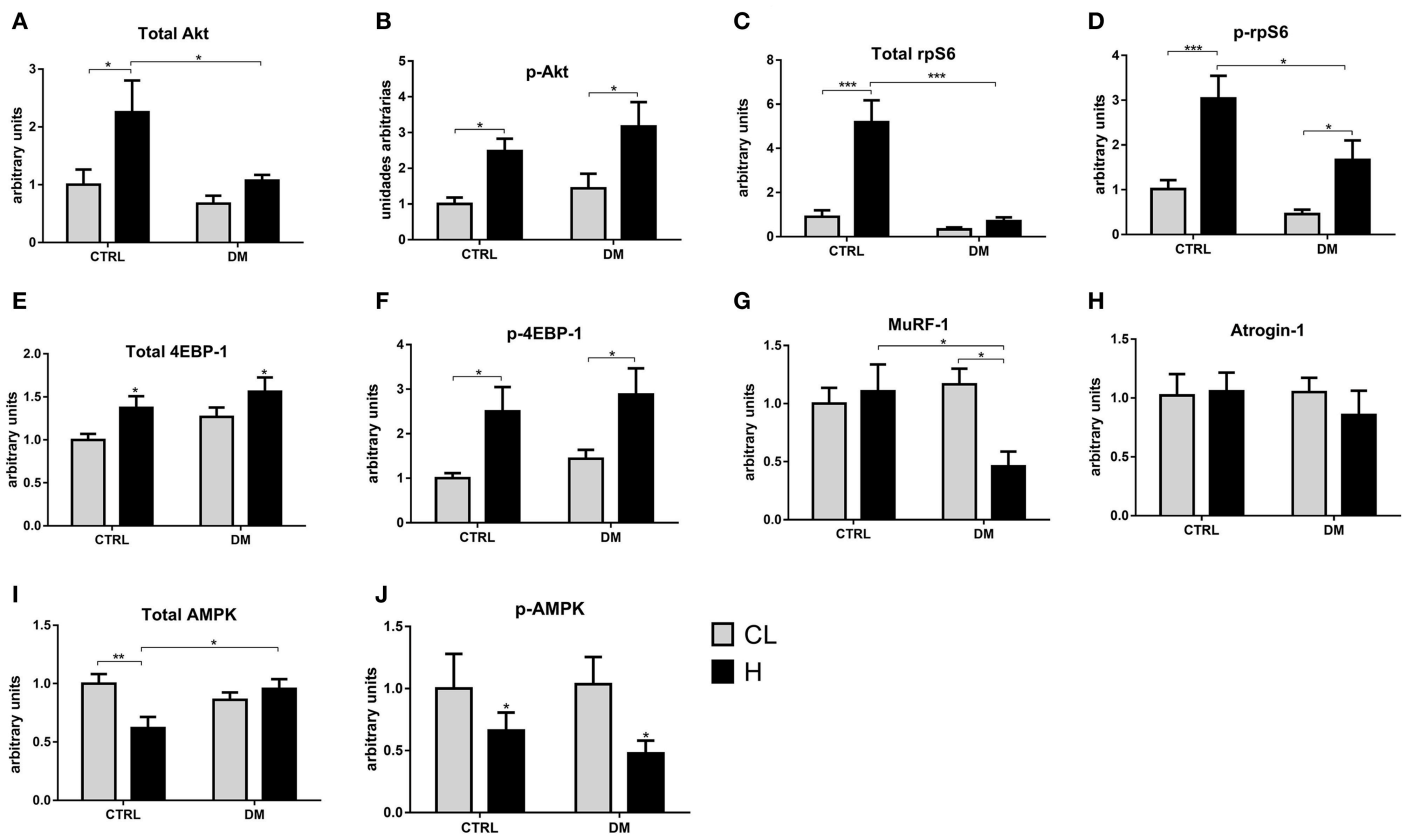
**(A–D, F–I)** Contents of signaling proteins associated with protein synthesis and degradation in the EDL muscle after 7 days of overload. Quantitative analysis of western blotting of total and phosphorylated Akt, rpS6, AMPK, MuRF-1 and atrogin-1 of the EDL muscle. Results are expressed as mean ± SEM of at least six animals. ^*^p < 0.05, ^**^p < 0.01, ^***^p < 0.001 using two-way ANOVA followed by Bonferroni post-test. In **(E,J)**, ^*^p < 0.05 CTRL-CL/DM-CL vs. CTRL-H/DM-H using two-way ANOVA only. CTRL, Control group; DM, Diabetic group; CL, Contralateral; H, Hypertrophy. Western blotting gels and ponceau staining are presented in Supplementary Material.

There was an increase in the content of total Akt by 2.2-fold in the EDL muscle of the control group after 7 days of overload. The content of p-Akt^Ser473^ was increased by 2.5-fold in the control and in the diabetic groups. Total rpS6 content was increased by 4.2-fold in the control group but did not change in the diabetic rats. The content of p-rpS6^Ser244/240^ was increased by 3.2-fold in the control group and by 2.6-fold in the diabetic due to overload stimulus. The total 4EBP-1 content, after 7 days of overload, was increased by 37% in the control group and by 23% in the diabetic. The contents of p-4EBP-1^Thr37/46^ was increased by 2.5-fold in the control group and by 2-fold in diabetic rats. The total AMPK content in the control group submitted to overload was reduced by 38%. The p-AMPK^Thr172^ content was decreased by 34% in the control and by 54% in the diabetic group submitted to overload. The content of MuRF-1 changed only in the diabetic group submitted to overload with a 51% decrease. Quantitative analysis of western blots after 7 days of overload in the EDL muscle is in Figure 4.

**Figure 5:**
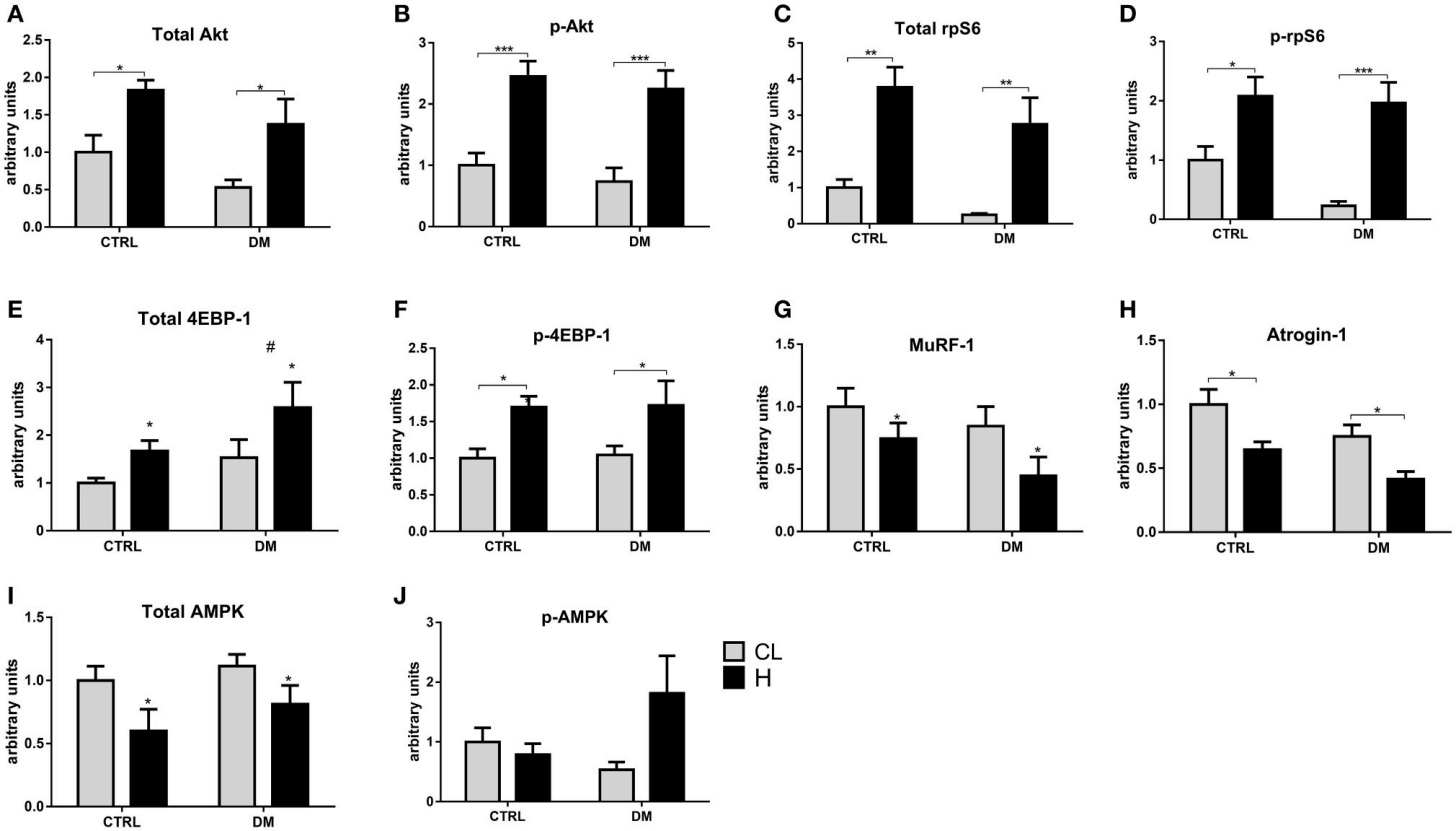
**(A–D, F,H,J)** Contents of signaling proteins associated with protein synthesis and degradation in the soleus muscle after 7 days of overload. Quantitative analysis of western blotting of total and phosphorylated Akt, rpS6, AMPK, MuRF-1 and atrogin-1 of the soleus muscle. Data are expressed as mean ± SEM of at least six animals. ^*^p < 0.05, ^**^p < 0.01, ^***^p < 0.001 using two-way ANOVA followed by Bonferroni post-test. In **(E)**, ^#^*p* < 0.05 for CTRL vs. DM and ^*^p < 0.05 for CTRL-CL/DM-CL vs. CTRL-H/DM-H, in **(G)**, ^*^p < 0.05 for CTRL-CL/DM-CL vs. CTRL-H/DM-H, in **(I)**, ^*^p < 0.05 for CTRL-CL/DM-CL vs. CTRL-H/DM-H using two-way ANOVA only. Control group; DM, Diabetic group; CL, Contralateral muscle; H, Hypertrophied muscle. Western blotting gels and ponceau staining are presented in Supplementary Material.

The total Akt content in the soleus muscle after 7 days of overload in the control group was increased by 83% whereas in the diabetic rats there was a 2.6-fold increase (Figure 5A). The content of p-AKT^Ser473^ increased by 2.4-fold in the control group whereas in diabetic rats there was a 3-fold increase (Figure 5B). The total rpS6 content increased by 3.7-fold (Figure 5C) in the control group and by 11-fold in the diabetic group. The contents of p-rpS6^Ser244/240^ increased by 2-fold in the control group and by 8.6-fold in the diabetic rats due to hypertrophy (Figure 5D). The total 4EBP-1 content, after 7 days of overload, increased by 68% (Figure 5E) in the control and in the diabetic groups. The contents of p-4EBP-1^Thr37/46^ in the soleus muscle increased (by 67%) in the control and in the diabetic groups (Figure 5F). The content of MuRF-1 decreased by 26% (Figure 5G) due to soleus muscle hypertrophy in the control and by 47% in the diabetic groups. Atrogin-1 had 36% decrease in the control and 45% reduction in the diabetic group (Figure 5H). The total AMPK content in the soleus muscle of the control group submitted to overload reduced by 40% and in the diabetic group by 27% (Figure 5I).

### Modulation of mRNA content after overload

**Figure 6:**
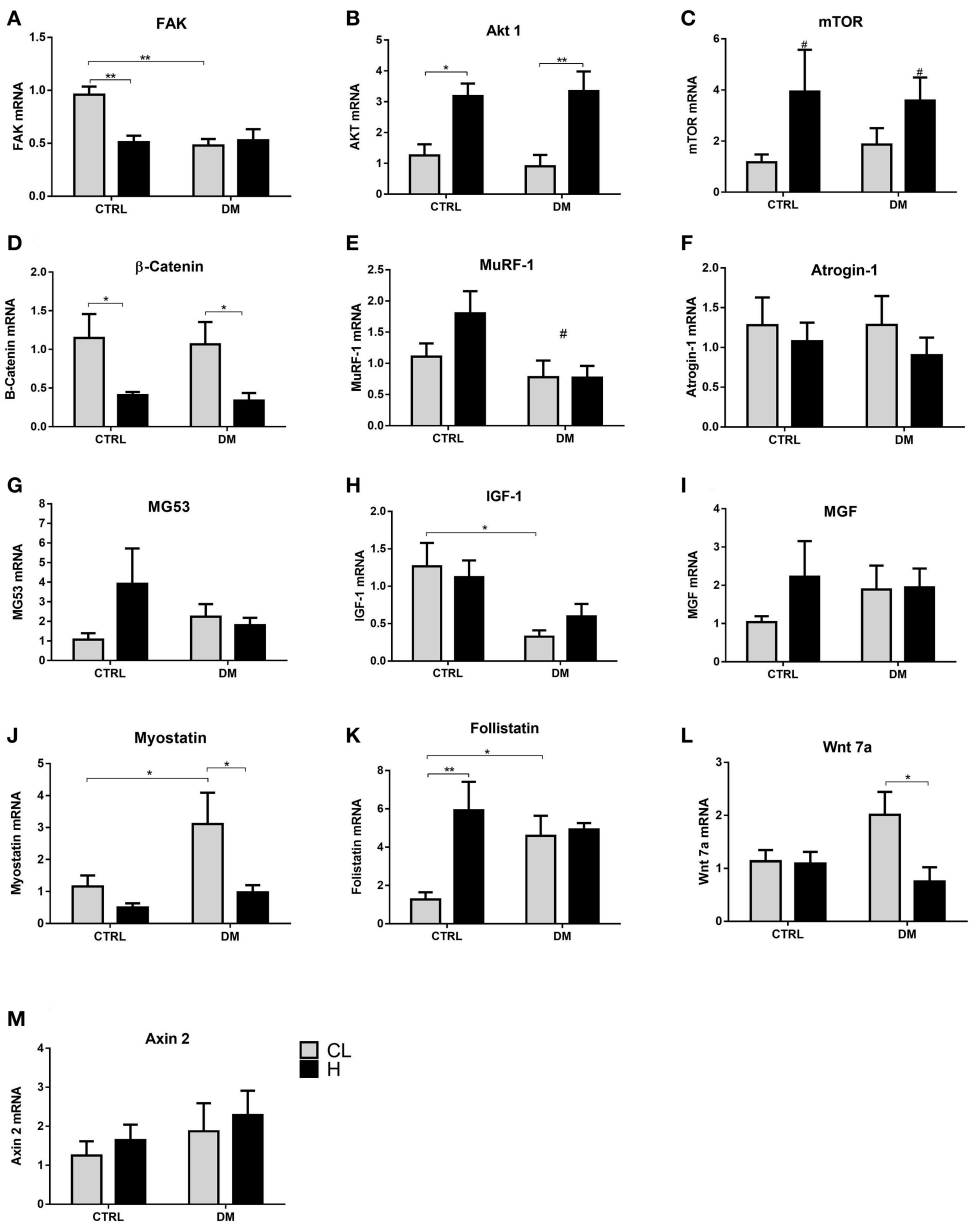
**(A,B,D, F–M)** mRNA expression of several genes in the EDL muscle after 7 days of overload. Results are presented as mean ± SEM of at least six animals. Results were analyzed using two-way ANOVA followed by Bonferroni post-test. ^*^p < 0.05, ^**^p < 0.01. In **(C)**, ^#^*p* < 0.05 for CTRL-CL/DM-CL vs. CTRL-H/DM-H, in **(E)**, ^#^*p* < 0.05 for CTRL vs. DM using two-way ANOVA only. CL, Contralateral muscle; H, Hypertrophied muscle; DM, Diabetic group; CTRL, Control group.

In the control group, regarding the hypertrophic response, the amount of mRNA was different for the genes: FAK, 46% reduction in expression; Akt1, 2.5-fold increase; mTOR, 3.3-fold increase; β-catenin, 64% reduction; follistatin, 4.6-fold increase (Figure 6). In the diabetic group submitted to overload, mRNA response was different for the following genes: Akt1, increase of 3.6-fold; mTOR, 92% increase; β-catenin and myostatin, both 68% reduction; Wnt7a, reduction of 62%.

mRNA expression in the contralateral EDL muscle was different between the control and the diabetic groups for the following genes: FAK, 49% lower in the diabetic group; IGF-1, 74% lower in the diabetic group; myostatin, 2.6-fold higher in the diabetic group; follistatin, 3.6-fold higher in the diabetic group. mTOR increased upon hypertrophy only in both groups (by 2.3-fold in CTRL and by 92% in DM) and MuRF-1 decreased due to diabetes (by 29% in the contralateral muscle and by 57% in the hypertrophied muscle).

**Figure 7:**
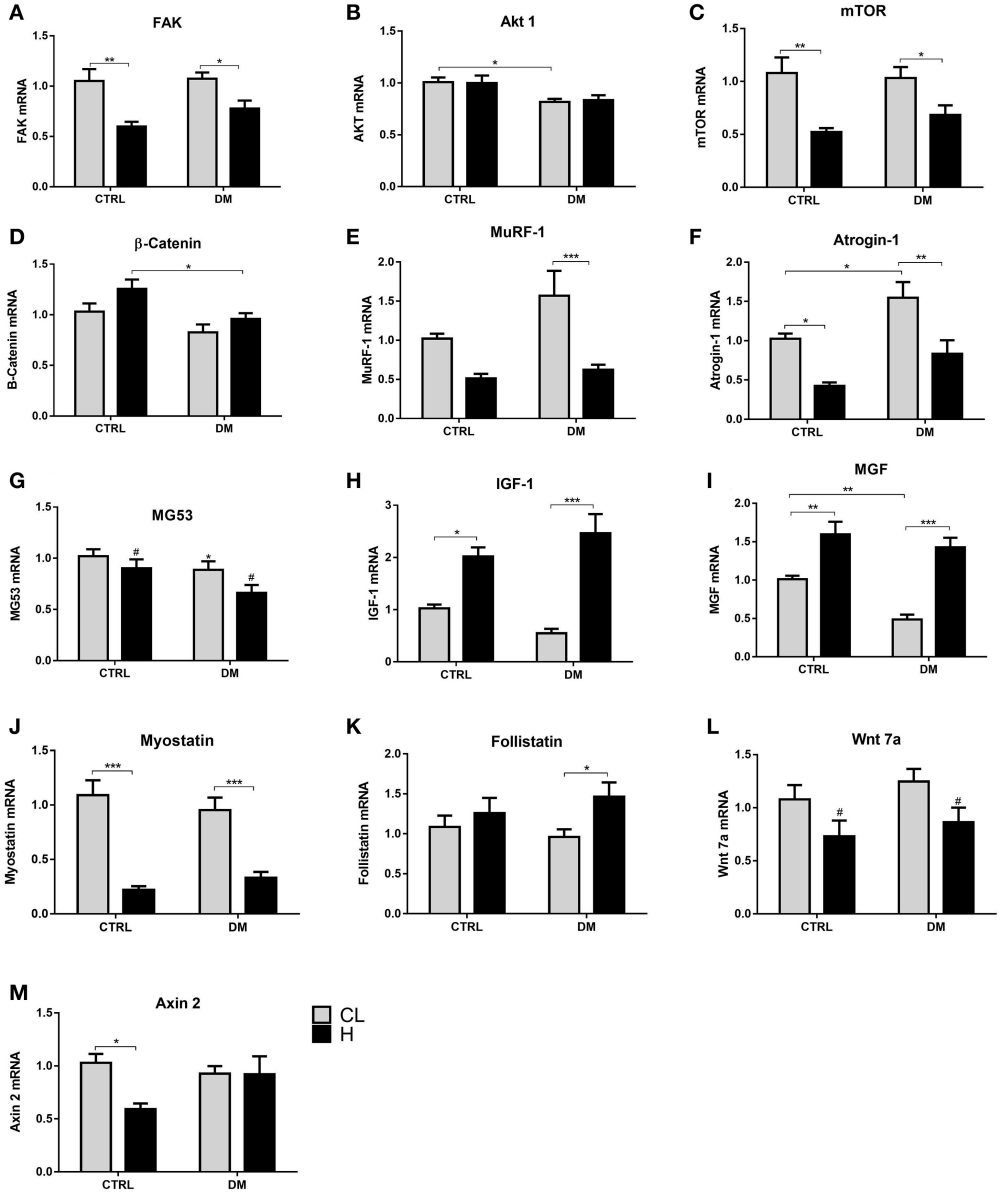
**(A–F, H–K, M)** mRNA expression of several genes in the soleus muscle after 7 days of overload. Results are presented as mean ± SEM of at least nine animals. Data were analyzed using two-way ANOVA followed by Bonferroni post-test. ^*^p < 0.05, ^**^p < 0.01, ^***^p < 0.001. In **(G)**, ^*^p < 0.05 CTRL vs. DM and ^#^*p* < 0.05 for CTRL-CL/DM-CL vs. CTRL-H/DM-H, in **(L)**, ^#^*p* < 0.05 for CTRL-CL/DM-CL vs. CTRL-H/DM-H using two-way ANOVA only. CL, Contralateral muscle; H, Hypertrophied muscle; DM, Diabetic group; CTRL, Control group.

The mRNA expression was also assessed after 7 days of overload in the soleus muscle (Figure 7). Regarding the hypertrophic response, the amount of mRNA in the control group was different for the genes: FAK, 43% reduction; mTOR, 51% reduction; Atrogin-1, 58% reduction; MG53, 11% reduction; IGF-1, 98% increase; MGF, 57% increase; myostatin, 79% reduction; Wnt7a, 33% reduction, axin2, 42% reduction.

In the diabetic group upon hypertrophy, the amount of mRNA was different for the following genes: FAK, 27% reduction; mTOR, 33% reduction; MuRF-1, reduction by 60%; Atrogin-1, 45% reduction; MG53, reduction of 25%; IGF-1, 4.5-fold increase; MGF, 2.9-fold increase; myostatin, reduction by 65%; follistatin, 52% increase; Wnt7a, reduction of 30%. MG53 presented a decrease due to diabetes only (by 14% in the contralateral muscle and by 26% in the hypertrophied muscle).

The mRNA expression in the hypertrophied soleus muscle was different between the control and diabetic groups for the following genes: β-catenin, 23% lower in the diabetic group. mRNA content in the contralateral soleus was different between the control and diabetic groups for the following genes: Akt 1, 19% lower in diabetic group; Atrogin-1, 50% higher in the diabetic group; MGF, 51% lower in the diabetic group.

## Discussion

In our previous study, EDL and soleus muscles (exhibiting a myopathy condition) of 30-days streptozotocin-induced diabetic rats had similar response to hypertrophic stimulus as the control animals (Fortes et al., 2015). Our previous work was focused on a skeletal muscle hypertrophic response after an already established chronic diabetic condition (in diabetic myopathy) using two different protocols. First, animals were rendered diabetic and remained hyperglycemic for 30 days before being submitted to overload-induced hypertrophy for 7 days. Second, in a different set of animals, diabetes was induced for 30 days before overload-induced hypertrophy for another 30 days period. In the present study, we investigated the mechanisms associated to overload-induced muscle hypertrophy and tested if the hypertrophy would be able to counteract the hypotrophy observed at the onset of type I diabetes (before diabetic myopathy manifestation).

Several signaling pathways control protein synthesis and degradation act synergistically during hypertrophic stimulation. We reported in the previous study that the contribution of each pathway in the control of muscle mass varies between the control and diabetic groups. The diabetic rats had to overcome diabetic myopathy and the consequences of low plasma insulin levels to ensure skeletal muscle mass gain. The differences between groups may be also associated with the muscle recruitment trend and the susceptibility of each muscle to the metabolic alterations caused by the disease, and the distinct composition of fiber types such as in the soleus muscle (predominantly type I) and EDL (predominantly type II) muscles (Cotter et al., 1989). Although the response to overload was comparable to control, the hypertrophic response in diabetic animals was not able to restore muscle mass to control values (of the contralateral muscle), which could be related to the onset of hypertrophic stimulation that occurs after a severe hypotrophy caused by chronic diabetes. In the present study, the hypertrophic stimulus was initiated concomitantly with the diabetes induction. The purpose was to promote hypertrophic stimulation in the early stages of diabetes, when protein degradation signaling is more intense, as an attempt to prevent diabetes-induced muscle hypotrophy.

**Figure 8:**
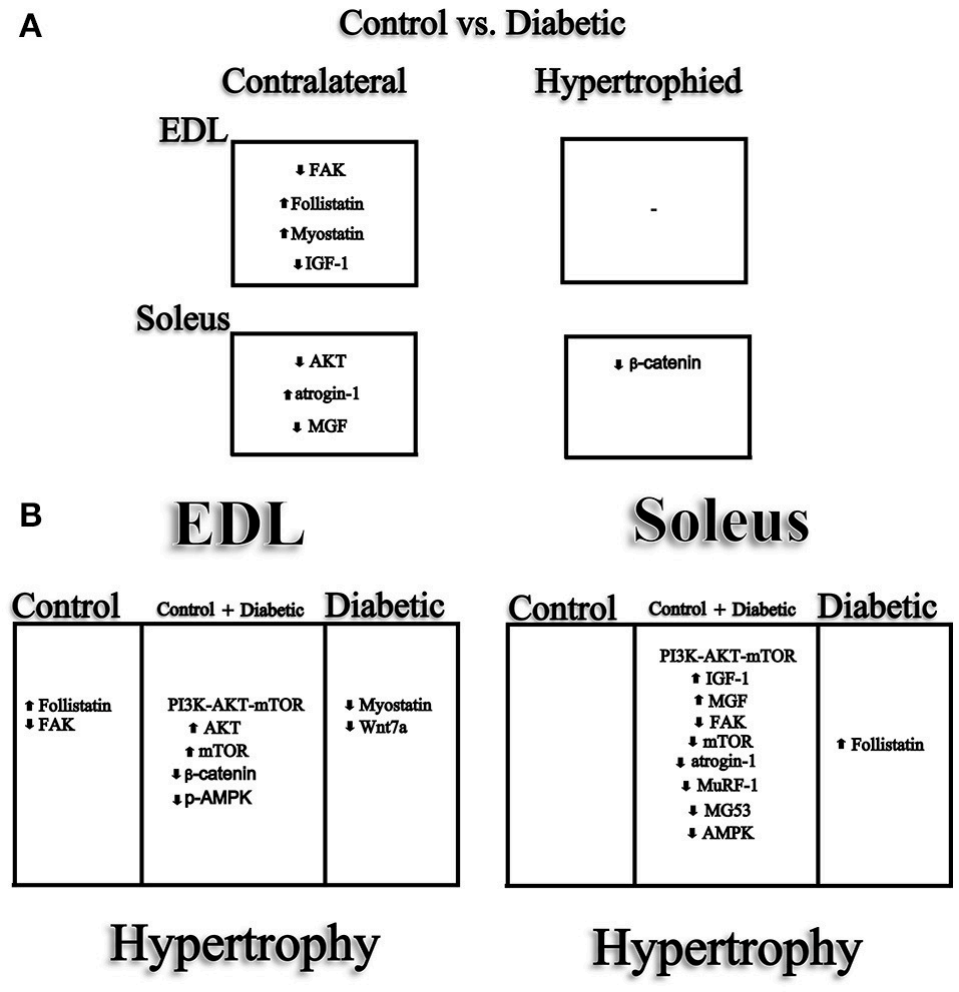
Main signaling pathways involved in the hypertrophy of the soleus and EDL muscles. **(A)** Effect of the early diabetic condition upon mRNA content and **(B)** modulation of the main signaling pathways involved in the hypertrophy of the soleus and EDL muscles in control or diabetic animals and in both.

The overload maintained soleus muscle mass, CSA of the muscle fibers and force production in diabetic rats to similar values or even higher than in the contralateral muscle of control animals. The EDL muscles of diabetic animals were not able to reach the absolute contralateral values of the control group even after 30 days of hypertrophic stimulus. The greater response of the soleus muscle may be related to activation of different signaling pathways of protein synthesis and degradation as compared to EDL (Figure 8). EDL muscle is more susceptible to force and muscle mass loss than soleus muscle (Paulus and Grossie, 1983). The EDL muscle of diabetic rats suffered greater hypotrophy than the soleus muscle. A more severe hypotrophy in the glycolytic/fast-twitch fibers may be a result of the effects of glucocorticoids; the plasma levels of corticosterone are elevated in streptozotocin-induced diabetic rats (Rhees et al., 1983) and these are the most susceptible fibers to the catabolic action of this hormone (Goldberg and Goodman, 1969). Reactive oxygen species (ROS) have been reported to, in long-term along with advanced glycation end-products (AGEs), promote skeletal muscle loss especially in the diabetic condition through a tightly coupling with PI3K/AKT signaling pathway (Grzelkowska-Kowalczyk et al., 2013; Zuo and Pannell, 2015; Zuo et al., 2015). Diabetic oxidative stress also impairs protein turnover and apoptotic process leading to muscle loss (Powers et al., 2012). Signaling differences (Figure 8A) might play an important role in the more pronounced atrophy of the EDL muscle reported.

The signaling pathway involved in the soleus muscle hypertrophy in diabetes was comparable to that of the control rats. The same was not observed for EDL muscle hypertrophy that may have contributed to the lower hypertrophic response in the diabetic group. mRNA expression of IGF-1 and MGF in the EDL muscle did not respond to hypertrophic stimulus in diabetic animals as observed in the soleus muscle. This may have contributed for the low increase in the phosphorylated and total rpS6 contents and for the lack of responsiveness of atrogin-1 expression, which was not reduced as in the soleus due to hypertrophic stimulation. In the EDL muscle, there was a reduction of myostatin, whereas in the soleus a reduction of myostatin concomitantly with an increase in follistatin was described. Therefore, the restoration of muscle mass for values similar to the contralateral control was far more effective in the soleus.

IGF stimulates protein synthesis during overload and promotes hypertrophy (Coleman et al., 1995; Bodine et al., 2001; Schiaffino and Mammucari, 2011). However, overload-induced hypertrophy does not depend exclusively on the IGF-1 pathway stimulus since it occurs even when IGF-1 receptors are inactivated (Spangenburg et al., 2008; Klossner et al., 2009). IGF-1 activates the PI3K/Akt/mTOR pathway and its main effectors (p70S6K, rpS6, 4EBP-1) that induce synthesis of myofibrillar proteins (Bodine et al., 2001; Richter and Sonenberg, 2005; Bodine, 2006). Seven days of soleus muscle overload promoted an increase of IGF-1 and MGF mRNA expression in the diabetic and the control groups. In animals submitted to 37 days of diabetes, the MGF mRNA expression in the hypertrophied soleus muscle was also markedly elevated (Fortes et al., 2015). IGF-1 expression was lowered in the EDL muscle of the diabetic group even after 7 days of overload. As mentioned above, it may have contributed to the reduced hypertrophic response of the EDL muscle in diabetic animals when compared to the soleus. The lack of IGF-1/MGF response might be associated with the severe limitation for the muscle mass maintenance in diabetic condition. In animals submitted to 37 days of diabetes, IGF-1 mRNA expression in the hypertrophied EDL muscle was markedly elevated (about 10-fold in the control and 5-fold in the diabetic groups) (Fortes et al., 2015). This difference between soleus and EDL muscles responses in our previous study indicates that both the period that the muscle remains under mechanical stimulation and the long-term diabetic myopathy regulate the gene expression of growth factors when compared to the initial phase diabetes onset. The diabetic condition affects the skeletal muscle, hypertrophic response, the susceptibility to contraction-induced injury, the quantity and activation of satellite cells, and the amount and composition of extracellular matrix (Brannon et al., 1989; Umpierrez et al., 1989; Gulati and Swamy, 1991; Law et al., 2012; D'Souza et al., 2016). Protein synthesis signaling reaches the activation peak within 7 days of muscle overload regardless hormonal stimulation does not follow the same time course (Armstrong and Ianuzzo, 1977; Farrell et al., 1999; Katta et al., 2010), indicating p-Akt and p-rpS6 protein levels were increased in the EDL and soleus muscles when submitted to 7 days of overload as also observed by others (Thomson and Gordon, 2006; Hamilton et al., 2010; Miyazaki et al., 2011). There was an increase in the total and phosphorylated content of 4EBP-1 protein upon hypertrophic stimulus corroborating previous studies (Bodine, 2006; Potter et al., 2013). Akt and mTOR, important components of the canonical pathway of protein synthesis, had different mRNA expression in the soleus and EDL muscles. Despite phosphorylation and total amount of Akt being higher in the hypertrophied control and diabetic groups, as also reported by others (Miyazaki et al., 2011; Fortes et al., 2015), mRNA expression changes did not closely follow the changes of protein content in the soleus muscle, which indicates posttranslational modifications (Ohlendieck, 2011, 2013; Lourenço dos Santos et al., 2015; Wende, 2016).

AMPK may decrease mTORC1 activity by phosphorylation of Tuberin (TSC2) in Thr1345 (Inoki et al., 2003) or raptor in Ser792 (Gwinn et al., 2008). Mounier et al. (2009), after AMPKα1 gene ablation, reported more pronounced overload-induced hypertrophy in plantar muscle (Mounier et al., 2009). Increased AMPK activation in the plantar muscle (McGee et al., 2008; Hamilton et al., 2014) associated with a decrease in skeletal muscle hypertrophy (Bolster et al., 2002; Thomson and Gordon, 2005) have also been described. We reported a more pronounced response of the PI3K/Akt/mTOR pathway in the soleus muscle in control and diabetic animals, despite not observing a reduction on the activation of AMPK. The AMPK activation may not be a determinant inhibitor of protein synthesis under the conditions of this study.

As a result of the PI3K/Akt/mTOR pathway activation, the MuRF-1, and atrogin-1 mRNA expression was inhibited by their upstream signaling protein, FOXO (Stitt et al., 2004), reducing proteasome-associated protein degradation. MuRF-1 and atrogin-1 might also affect skeletal muscle remodeling that occurs during growth (Baehr et al., 2014). MuRF-1 and atrogin-1 expression was decreased in the soleus muscle of the control and diabetic rats upon overload, and the same did not occur in the EDL muscle. This may be due to the lower activation of PI3K/Akt/mTOR pathway that contributed for the lower hypertrophic response in the EDL muscle.

MG53 expression was reduced by the diabetic condition as well by hypertrophic stimulus in the soleus muscle. This might be involved in the increased hypertrophic response of the soleus in diabetic animals as compared with the EDL muscle. MG53 is involved in the regulation of myogenic negative feedback and cleaves the insulin receptor substrate 1 (IRS-1) (Jung and Ko, 2010; Lee et al., 2010). MG53 acts synergistically with other two myogenic regulatory factors (MyoD—myogenic differentiation factor and MEF2—muscle enhancer factor 2) (Jung and Ko, 2010; Lee et al., 2010). MG53 promotes ubiquitination followed by degradation of IRS-1 and acts on the PI3K/Akt/mTOR signaling causing deficient synthesis of contractile myofibrillar proteins (Yi et al., 2013).

The expression of FAK, an enzyme involved in the mechanical signaling associated with skeletal muscle hypertrophy (Flück et al., 1999; Klossner et al., 2009), was increased in hypertrophied soleus and EDL muscles after 7 days of overload when animals were previously submitted to 30 days of diabetes (Fortes et al., 2015). However, when animals were submitted to a period of 7 days of overload initiated at the onset of diabetes, FAK mRNA expression had a decrease in both soleus and EDL muscles. These findings indicate that the expressions of proteins related to muscle hypertrophy changes with the period of the diabetic state.

The balance between myostatin and follistatin expressions also regulate skeletal muscle hypertrophy, with myostatin inhibiting and follistatin stimulating this process (Gilson et al., 2009). The absence of myostatin induces muscle growth whereas the elevation of its mRNA expression and exogenous administration causes muscle hypotrophy (McPherron and Lee, 1997; McPherron et al., 1997; Schuelke et al., 2004; Rodriguez et al., 2014). We reported a reduction in the myostatin mRNA in the EDL muscle. However, in the soleus, in addition to the reduction in myostatin, there was also an increase in follistatin mRNA. This combined response may contribute to a greater hypertrophic response in the soleus muscle in diabetes.

Wnt, β-catenin and axin influence skeletal muscle myogenesis, being necessary for skeletal muscle repair in response to injury (Newmire and Willoughby, 2015; Huraskin et al., 2016; Rudolf et al., 2016). There was no similar response of these three genes in the present study. The expression of β-catenin in the EDL muscle was reduced upon hypertrophy, which did not occur in the soleus muscle. Increased expression of β-catenin also participated in the soleus muscle hypertrophy process (Armstrong and Esser, 2005; Armstrong et al., 2006). β-Catenin expression was increased in the soleus muscle and at the same time Wnt remained almost unaltered. Since PI3K/Akt/mTOR pathway is more active in the soleus, there may be a greater inhibition of GSK3β in this muscle, corroborating for the increase (or maintenance) in β-catenin levels and therefore contributing for the hypertrophic response (Jones et al., 2015).

## Concluding remarks

The soleus and EDL muscles from diabetic animals submitted to overload at the onset of the disease (before diabetic myopathy manifestation) exhibited similar hypertrophic response. The increase of muscle forces occurred at the same magnitude as the muscle hypertrophy.

In the EDL muscle of diabetic animals, overload promoted hypertrophy via mechanical action, which involved the PI3K/Akt/mTOR pathway, reduced AMPK activation and decreased myostatin expression.

Hypertrophy was more pronounced in the soleus muscle of diabetic animals, as compared with the EDL, maintaining the muscle mass and force to similar values of the contralateral control muscle. This may be due to a more preserved hypertrophic signaling, in relation to the control group, including a higher content and activation of rpS6 than in the EDL muscle, increased mRNA expression of IGF-1, MGF, and follistatin and decreased mRNA expression of myostatin, MuRF-1 and atrogin-1.

We concluded that resistance exercise is an ally for the prevention of diabetic myopathy (hypotrophy) and when initiated early in the diabetes progression its effectiveness might be greater.

## Author contributions

MF conceived of the study, carried out the molecular experiments, statistical analysis, conducted the animals' surgeries, interpreted the results, and wrote the manuscript. MS assisted in the molecular experiments. GM participated in the molecular studies, interpreted the results and assisted in the draft of the manuscript. KV assisted its design and coordination, interpreted the results and revised the manuscript for important intellectual content. CdJP participated in the interpretation of the results and revised for important intellectual content. RC revised for important intellectual content, assisted in the statistical analysis and draft the manuscript. All authors read and approved the final manuscript.

### Conflict of interest statement

The authors declare that the research was conducted in the absence of any commercial or financial relationships that could be construed as a potential conflict of interest.
