# Supplementary material for: Hypertrophy Stimulation at the Onset of Type I Diabetes Maintains the Soleus but Not the EDL Muscle Mass in Wistar Rats
Source: Front Physiol. 2017 Oct 26;8:830. doi: 10.3389/fphys.2017.00830 (PMC5662641; doi:10.3389/fphys.2017.00830)
Supplement: Supplementary file 1 [file DataSheet1.PDF]

OVERLOAD STIMULATION AT THE ONSET OF TYPE I DIABETES PRESERVES THE SOLEUS BUT NOT THE EDL MUSCLE MASS IN WISTAR RATS

Marco Aurélio Salomão Fortes[1,*]; Maria Vitória Martins Scervino[1]; Gabriel Nasri Marzuca-Nassr[1,2]; Kaio Fernando Vitzel[3]; Carlos Hermano da Justa Pinheiro[1] and Rui Curi[1,4]

1 Department of Physiology and Biophysics, Institute of Biomedical Sciences, University of São Paulo, Sao Paulo, Brazil

2 Department of Internal Medicine, Faculty of Medicine, Universidad de La Frontera, Temuco, Chile.

3 Massey Institute of Food Science and Technology, College of Health, Massey University, Albany, New Zealand

4 Interdisciplinary Post-Graduate Program in Health Sciences, Cruzeiro do Sul University, Sao Paulo, Brazil

*Corresponding author: Marco Aurélio Salomão Fortes, Department of Physiology and Biophysics, Institute of Biomedical Sciences, University of São Paulo, room 105, 1524 Prof. Lineu Prestes Av, Cidade Universitária, Butantã, São Paulo, SP - CEP:05508-000, Brazil. Tel: +55 11 3091 7245. E-mail: markofortes@hotmail.com

Supplementary Figures and Tables

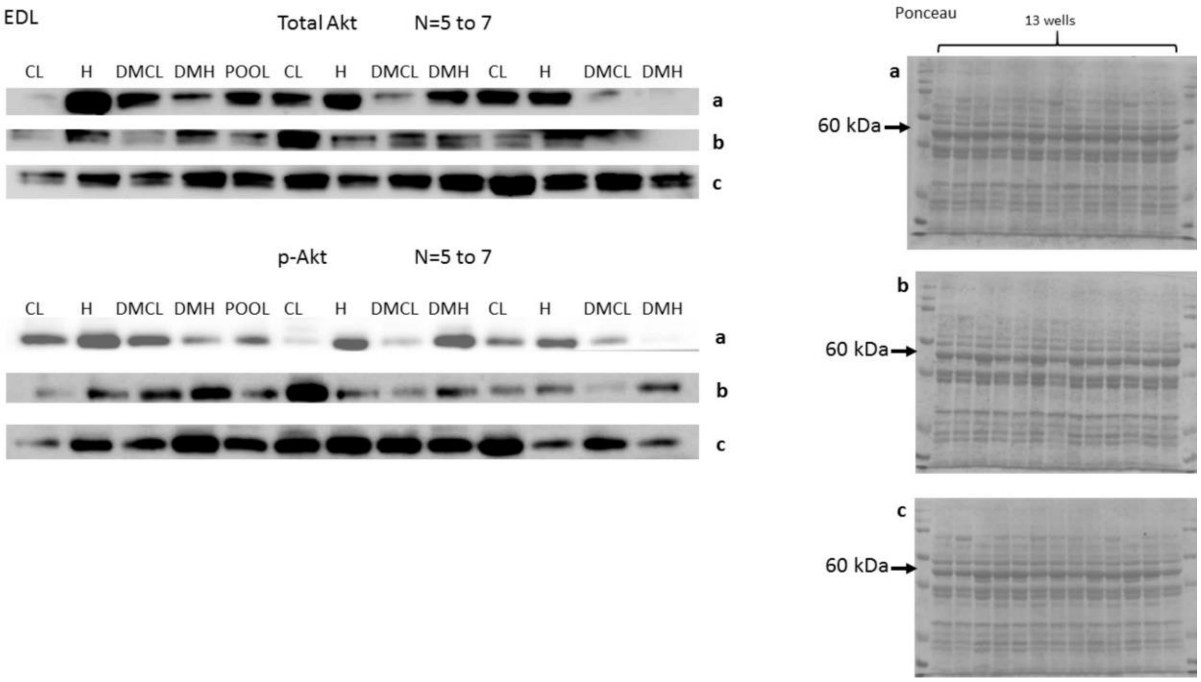

Supplementary Figure 1: Ponceau and gels obtained by western blotting of the total Akt protein and its phosphorylated form in the EDL muscle after 7 days of overload. 5 to 7 animals were used for the plot. DMCL – contralateral muscle diabetic group; DMH – hypertrophied muscle diabetic group; CL - contralateral; H – hypertrophy; P - pool containing a mixture with equal parts of all samples – used to normalize Ponceau S quantitative results.

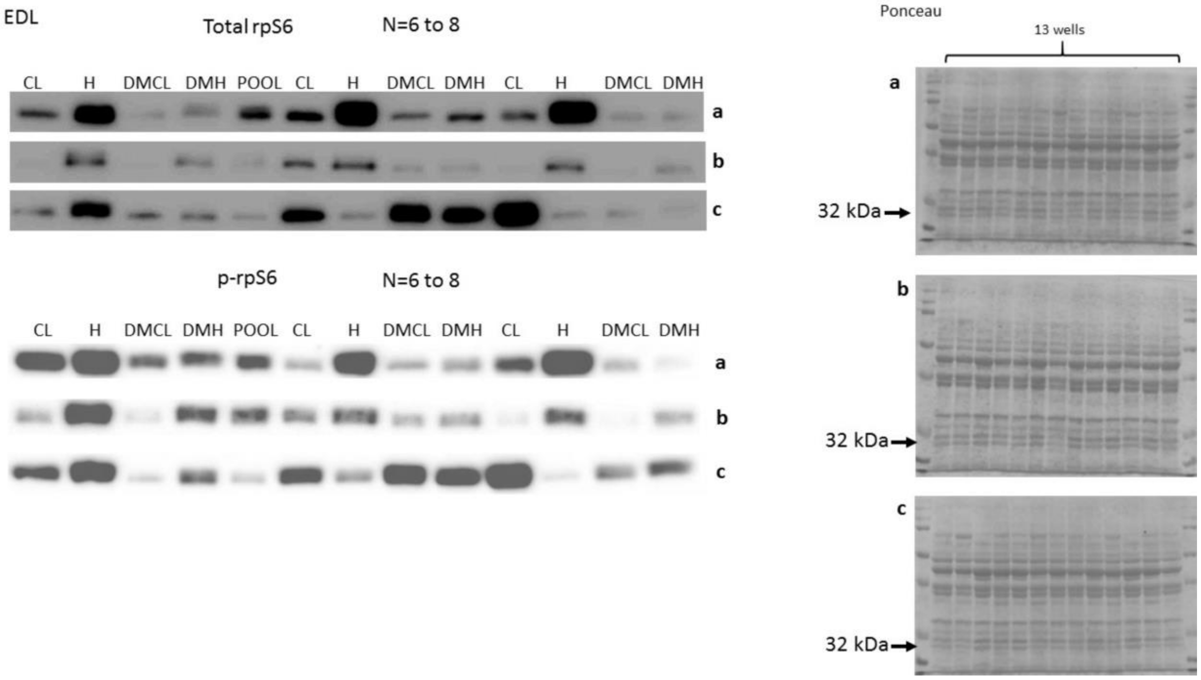

Supplementary Figure 2: Ponceau and gels obtained by western blotting of the total rpS6 protein and its phosphorylated form in the EDL muscle after 7 days of overload. 5 to 7 animals were used for the plot. DMCL – contralateral muscle of the diabetic group; DMH – hypertrophied muscle of the diabetic group;

CL - contralateral; H – hypertrophy; P - pool containing a mixture with equal parts of all samples – used to normalize Ponceau S quantitative results.

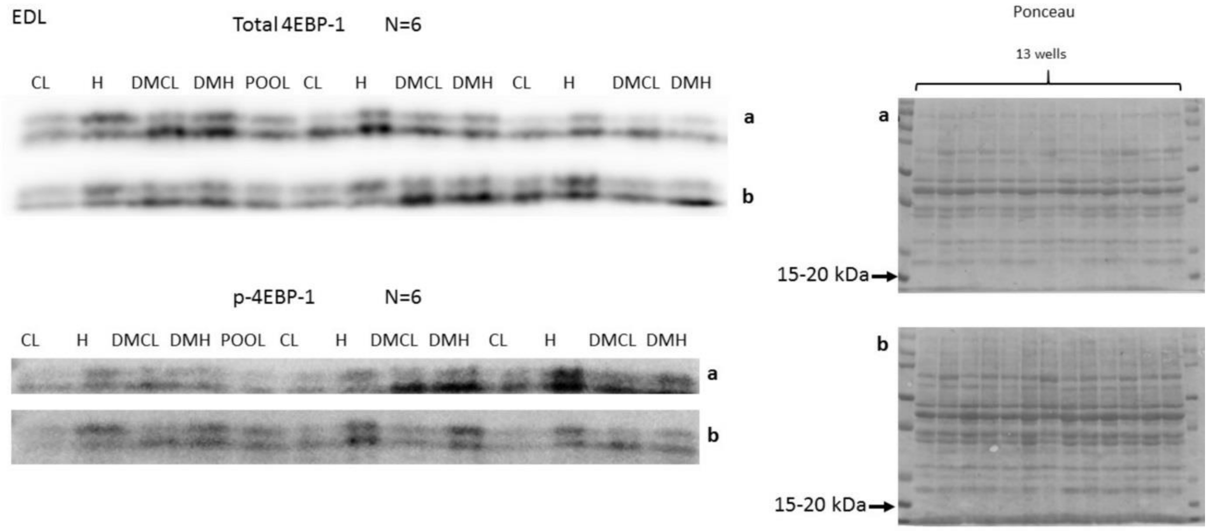

Supplementary Figure 3: Ponceau and gels obtained by western blotting of the total 4EBP-1 protein and its phosphorylated form in the EDL muscle after 7 days of overload. 5 to 7 animals were used for the plot. DMCL – contralateral muscle of the diabetic group; DMH – hypertrophied muscle of the diabetic group; CL - contralateral; H – hypertrophy; P - pool containing a mixture with equal parts of all samples – used to normalize Ponceau S quantitative results.

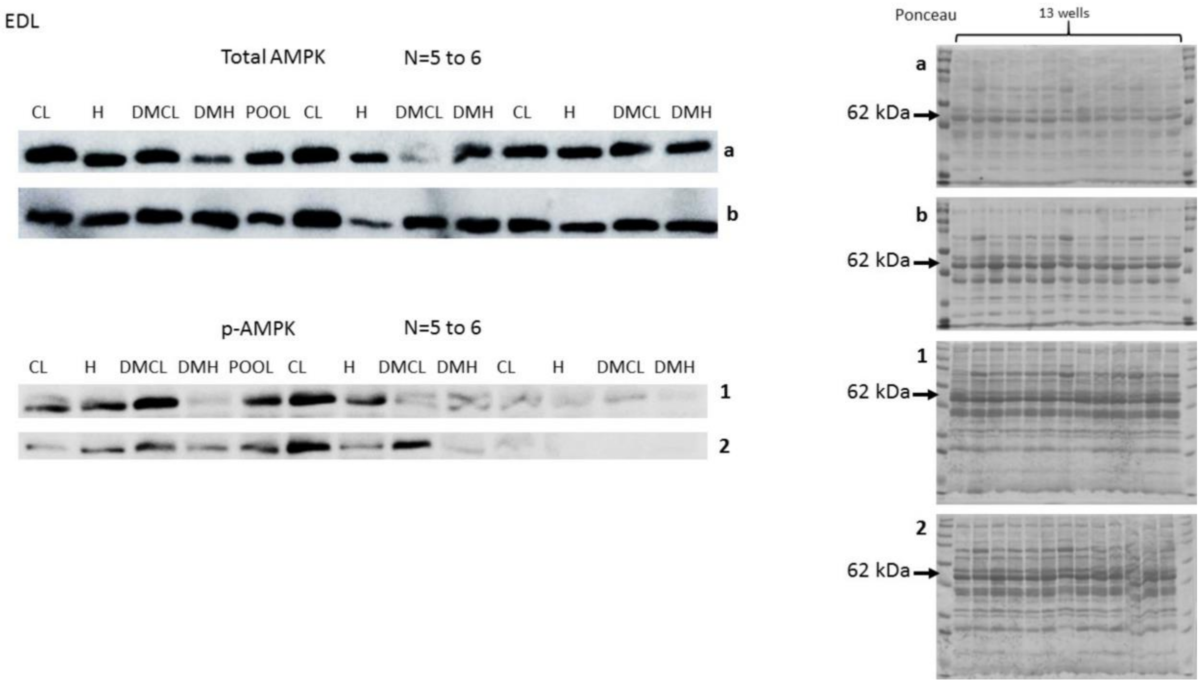

Supplementary Figure 4: Ponceau and gels obtained by western blotting of the total AMPKα1 protein and its phosphorylated form in the EDL muscle after 7 days of overload. 5 to 6 animals were used for the plot. DMCL – contralateral muscle of the diabetic group; DMH – hypertrophied muscle of the diabetic

group; CL - contralateral; H – hypertrophy; P - pool containing a mixture with equal parts of all samples – used to normalize Ponceau S quantitative results.

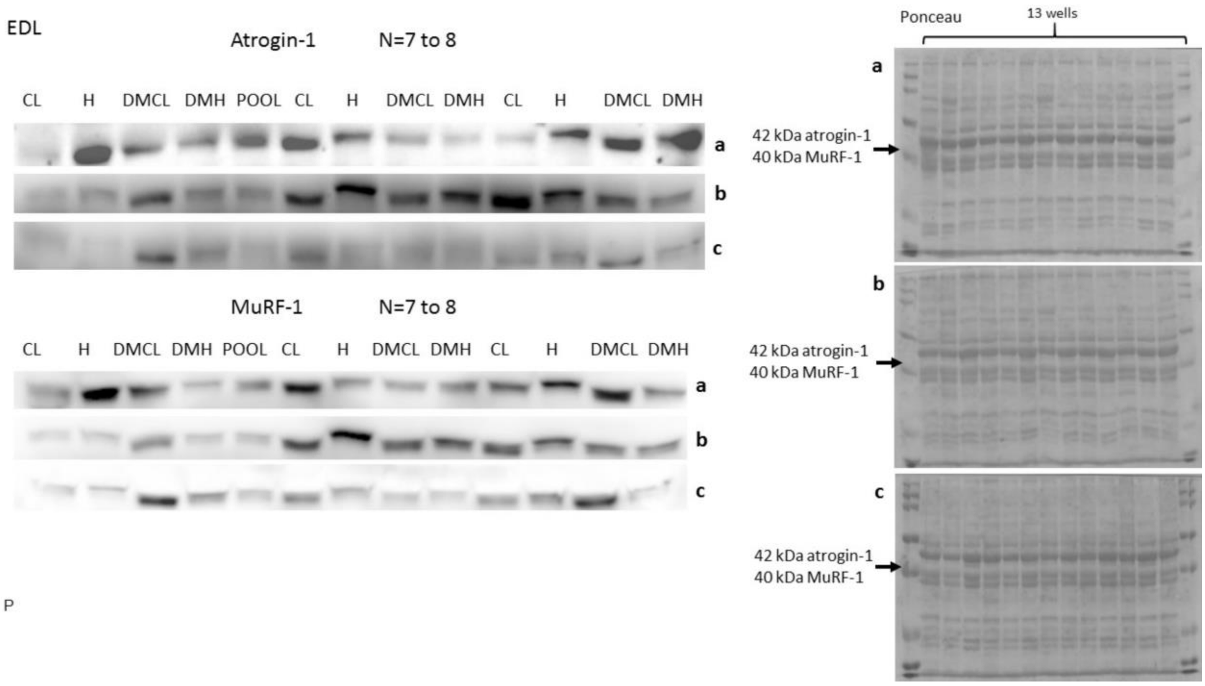

Supplementary Figure 5: Ponceau and gels obtained by western blotting of the MuRF-1 and atrogin-1 in the EDL muscle after 7 days of overload. 7 to 8 animals were used for the plot. DMCL – contralateral muscle of the diabetic group; DMH – hypertrophied muscle of the diabetic group; CL - contralateral; H – hypertrophy; P - pool containing a mixture with equal parts of all samples – used to normalize Ponceau S quantitative results.

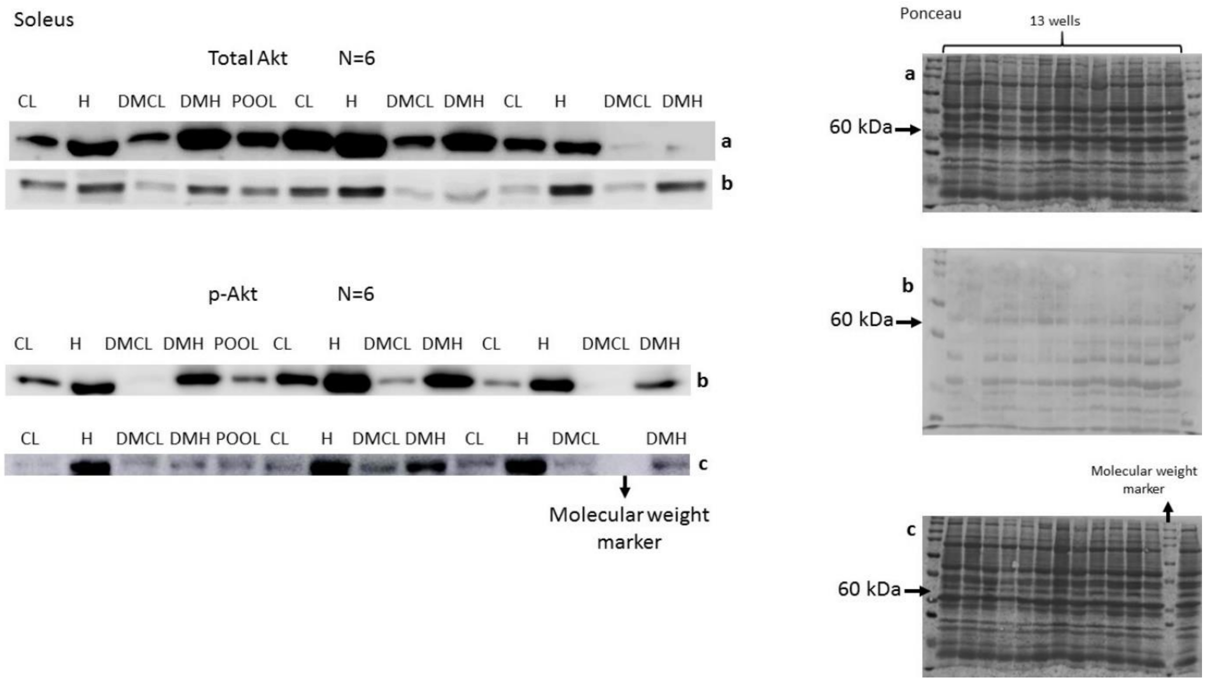

Supplementary Figure 6: Ponceau and gels obtained by western blotting of the total Akt protein and its phosphorylated form in the soleus muscle after 7 days of overload. 6 animals were used for the plot.

DMCL – contralateral muscle diabetic group; DMH – hypertrophied muscle diabetic group; CL - contralateral; H – hypertrophy; P - pool containing a mixture with equal parts of all samples – used to normalize Ponceau S quantitative results.

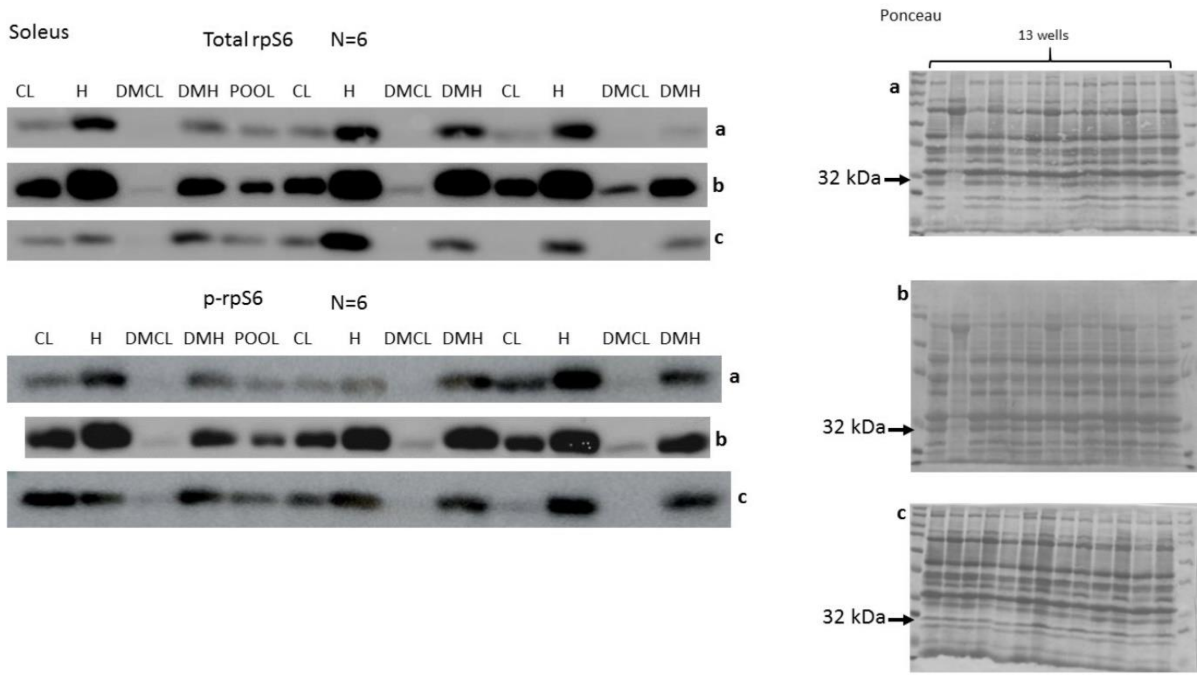

Supplementary Figure 7: Ponceau and gels obtained by western blotting of the total rpS6 protein and its phosphorylated form in the soleus muscle after 7 days of overload. 6 animals were used for the plot. DMCL – contralateral muscle of the diabetic group; DMH – hypertrophied muscle of the diabetic group; CL - contralateral; H – hypertrophy; P - pool containing a mixture with equal parts of all samples – used to normalize Ponceau S quantitative results.

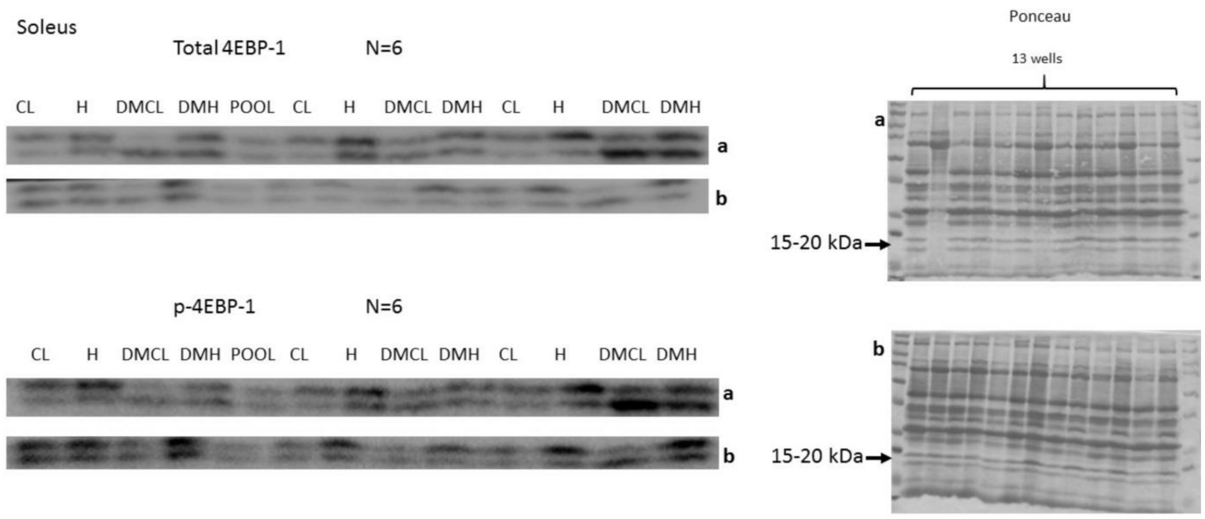

Supplementary Figure 8: Ponceau and gels obtained by western blotting of the total 4EBP-1 protein and its phosphorylated form in the soleus muscle after 7 days of overload. 6 animals were used for the plot. DMCL – contralateral muscle of the diabetic group; DMH – hypertrophied muscle of the diabetic group; CL - contralateral; H – hypertrophy; P - pool containing a mixture with equal parts of all samples – used to normalize Ponceau S quantitative results.

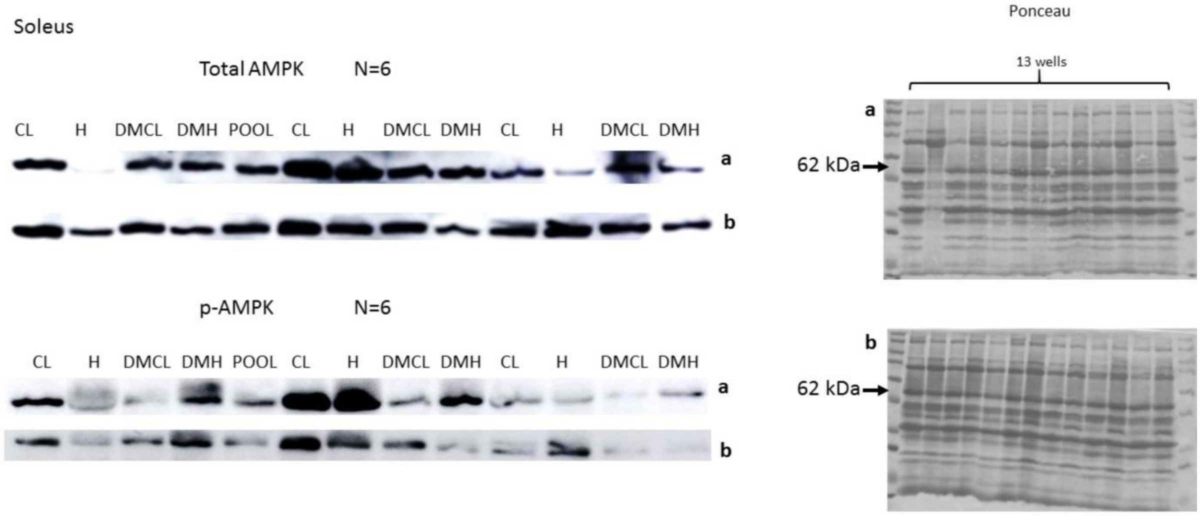

Supplementary Figure 9: Ponceau and gels obtained by western blotting of the total AMPKα1 protein and its phosphorylated form in the soleus muscle after 7 days of overload. 6 animals were used for the plot. DMCL – contralateral muscle of the diabetic group; DMH – hypertrophied muscle of the diabetic group; CL - contralateral; H – hypertrophy; P - pool containing a mixture with equal parts of all samples – used to normalize Ponceau S quantitative results.

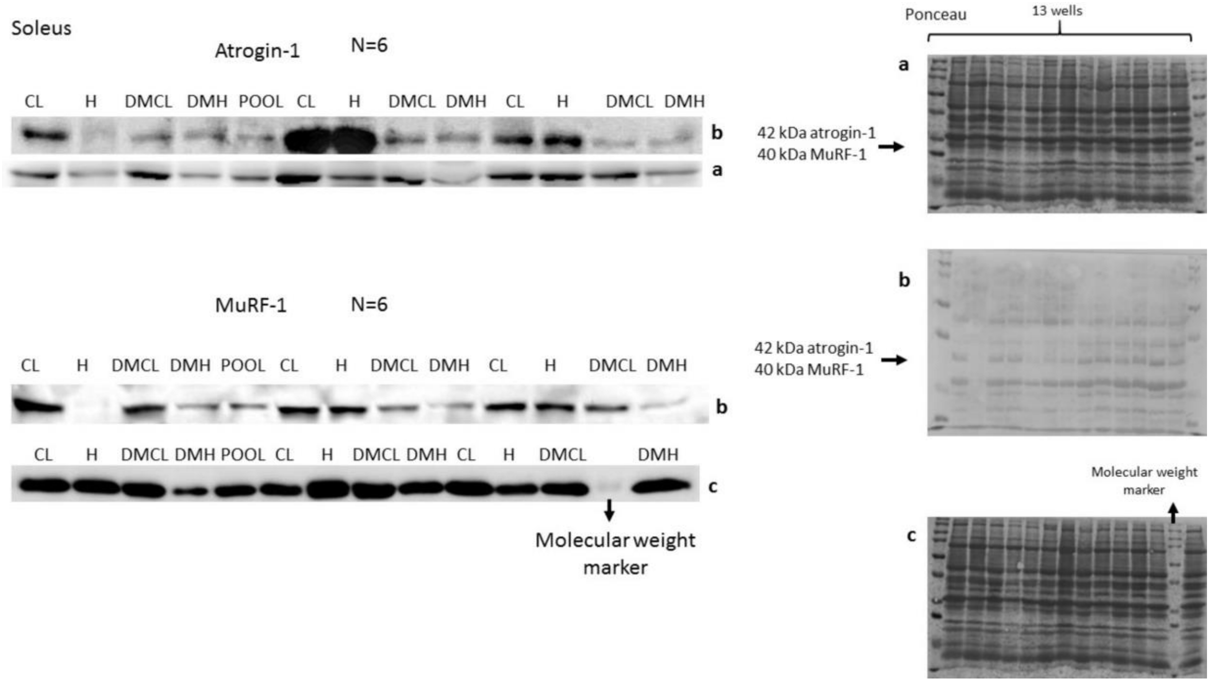

Supplementary Figure 10: Ponceau and gels obtained by western blotting of the MuRF-1 and atrogin-1 in the soleus muscle after 7 days of overload. 6 animals were used for the plot. DMCL – contralateral muscle of the diabetic group; DMH – hypertrophied muscle of the diabetic group; CL - contralateral; H – hypertrophy; P - pool containing a mixture with equal parts of all samples – used to normalize Ponceau S quantitative results.
